# Conformational Changes in the Structure of Dough and Bread Enriched with Pumpkin Seed Flour

**DOI:** 10.3390/plants11202762

**Published:** 2022-10-19

**Authors:** Svitlana Litvynchuk, Oleg Galenko, Alessio Cavicchi, Costanza Ceccanti, Chiara Mignani, Lucia Guidi, Anastasiia Shevchenko

**Affiliations:** 1 Department of Physics, National University of Food Technologies, 01601 Kyiv, Ukraine; 2 Department of Technology of Meat and Meat Products, National University of Food Technologies, 01601 Kyiv, Ukraine; 3 Department of Agriculture, Food and Environment, University of Pisa, 56124 Pisa, Italy; 4 Interdepartmental Research Center Nutrafood “Nutraceuticals and Food for Health”, University of Pisa, 56124 Pisa, Italy; 5 Department of Political Sciences, Communication and International Relations, University of Macerata, 62100 Macerata, Italy; 6 Department of Bakery and Confectionery Goods Technologies, National University of Food Technologies, 01601 Kyiv, Ukraine

**Keywords:** bread, dough, pumpkin seed flour, near-infrared reflection spectroscopy, protein

## Abstract

Pumpkin seed flour is a promising raw material for use in the technology of various bakery products. It has a high biological value and valuable amino acid profile. During the technological process of making bread, there are conformational changes in the protein structure. The purpose of the study was to determine the effect of pumpkin seed flour on conformational changes in the structure of protein substances of dough and bread from wheat flour by near-infrared reflection spectroscopy. The protein profile changed to complete when replacing 10% or more of wheat flour because the score for all amino acids was higher than 100%. The utilitarian coefficient indicates the same balance of amino acids in proteins of all samples. As the percentage of substitution increases, the number of amino acids used for anabolic purposes decreases, and these are more fully utilized by the body.

## 1. Introduction

During recent years, the increasing interest of consumers in fitness regimes and a healthy body appearance contributed to a rise in the demand for healthy products. Furthermore, after the spread of COVID-19, consumers were forced to change their attitude toward food; by staying at home, a lot of people enhanced awareness about food waste and a healthy lifestyle, adopting a positive adaptive behavior related to healthy diets and food systems [1]. According to Brigagão et al. [2], modern consumers have changed their food preferences regarding bioactive nutrient compounds of fruits and vegetables and their inedible parts.

Thus, among other recent trends, consumer attention to the nutraceutical aspect of food products is becoming mainstream, providing both challenges and opportunities to farmers and other actors involved in the agrifood supply chain: innovative “product-rich-in” and “free-from” became very profitable segments, offering, respectively, fortified/functional characteristics (for example, antioxidant properties, high content of proteins, prebiotics, probiotics, etc.), and products with a low or no content of particular attributes such as fat, sugar, lactose, gluten, etc.

Health problems can influence these food choices; however, an increasing number of people consume these products because of their linkage with a healthier lifestyle.

Several studies stress the effect of cognitive factors, socio-economic status, food-related values, and traits that influence the interest of consumers in functional food [3,4,5].

In this context, the trend of using non-traditional raw materials to increase the nutritional value of products and provide them with healthy properties is rising in the bakery industry [6]. Different types of flour (rice, soy, buckwheat, and others) are used to replace part of the wheat flour in the technology of wheat bread. Pumpkin seed flour has a valuable chemical composition of up to 45% protein and 15% fiber content [7]. It is also a source of other macro- and micronutrients, such as carbohydrates, vitamins, and minerals [8]. Pumpkin seed flour also has a high antioxidant capacity thanks to the high content of bioactive compounds such as tocopherols, carotenoids, provitamins, pigments, triterpenoids, phenolic compounds, and other derivatives [9,10].

In addition to the nutritional components, in the case of pumpkin seeds, another aspect to consider is the reduction of agroindustry waste provided by reusing seeds flour in preparing bread, cake, and other foodstuffs. An important challenge of the food industry is using alternative plant parts, such as peels and seeds, with a significant nutritional value in the formulation of food products, enriching their functional value and reducing costs in waste treatment [11]. Indeed, pumpkin seed flour is used in the technology of various bakery products. Given the increasing popularity of ready-to-eat bakery products such as pan and pita, the enrichment of these products with vegetables high in protein, fiber, and bioactive ingredients is relevant [12]. The composite flours (wheat flour and pumpkin seed flour) showed better water holding capacity and softer gels than mono-flour products [12]. Consistency and viscoelasticity were lower compared to samples of bread from only wheat flour. Dough strength and stability were reduced, lowering bread volume, but the nutritional quality improved, enhancing antioxidant activity [12].

The addition of pumpkin seed flour in the amount of 5–15% to whole wheat flour in the technology of bread increased crude protein, ash, and crude fat content, but the addition of higher levels of pumpkin seed flour might cause rancidity [13].

The size of the particles of pumpkin seed flour is larger than wheat wholemeal flour, and the water absorption capacity exceeds such an indicator for wheat flour by 1.5 times [14]. The gas-forming capacity of the dough with this additive decreased, as well as the amount of formed sugars, but, on the contrary, the amount of fermented sugars increased [14].

The increased amount of added partially defatted pumpkin seed flour (5–15%) decreased the water absorption capacity of the dough, and the dough stability increased from 9.43 to 10.23 min [15].

A key role in forming the structure of the dough belongs to the gliadin and glutenin fractions of protein, which, in the hydrated state, form gluten, a continuous viscoelastic network. In the technological process of making bread, there are molecular conformations of gluten proteins altering the viscoelastic properties of dough systems. They can be evaluated by numerous techniques, e.g., infrared spectroscopy [16].

Glutenins are multi-chained proteins, polymerized by disulfide bonds and not cross-linked. They provide gluten with elastic properties. Gliadin proteins contain intra-molecular disulfide bonds [17]. The breaking of them causes the unfolding of the protein molecules. They provide gluten with cohesive properties [18]. Proteins of pumpkin seed flour differ from proteins of wheat flour, so there are differences in dough behavior during fermentation.

The amino acid profile and protein digestibility in vitro provide the quality of proteins. The in vitro protein digestibility of pumpkin seed kernels is 90%, and that of defatted pumpkin seed flour is 77.91% [19].

In dough, there are microstructural and molecular changes in gluten proteins. The amorphous matrix of gluten at the microstructural level experienced a different degree of depolymerization into laminar and fibrillar structures. The development of these elements was the result of conformational changes in the secondary structure of gluten proteins [20]. Because of the absence of gluten in pumpkin seeds, the structure of proteins in flour with the addition of pumpkin seed flour changes during the breadmaking process, and differs from changes in wheat flour bread.

The aim of the study was to determine the effect of pumpkin seed flour on conformational changes in the structure of protein substances of dough and bread from wheat flour.

## 2. Results and Discussion

### 2.1. Protein and Amino Acid Composition and Functional Properties of Wheat and Pumpkin Seed Flours

**Figure 1:**
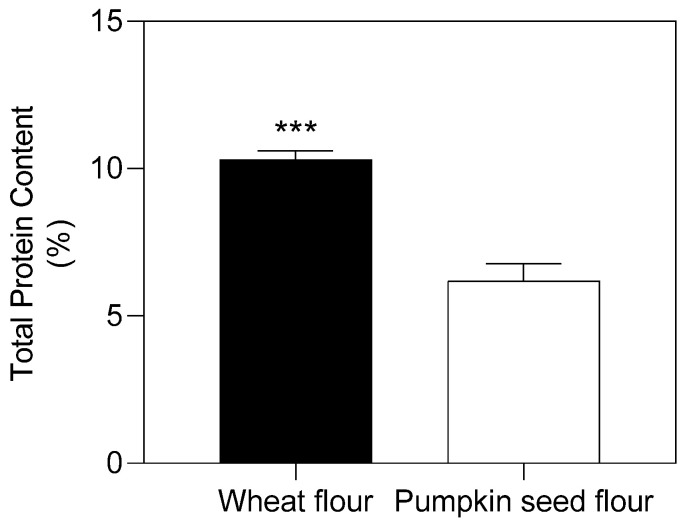
Total protein content (% dry mass) of wheat and pumpkin seed flour. Data were compared with Student’s *t*-test (*p* ≤ 0.05). Significance ***: *p* ≤ 0.001.

Flour plays a decisive role in forming the properties of dough for bakery products, especially for its protein content and composition [21]. The total content of proteins found in pumpkin seed flour was 40% lower than that of wheat flour (Figure 1).

Indeed, through the analysis of the fractional composition of proteins, higher albumin, glutenin, prolamin, and insoluble protein content in wheat flour was observed when compared with the pumpkin seed flour (Table 1). A higher presence of glutenin (gluten protein) in wheat flour than in pumpkin seed flour could represent a source of heterogeneity in the dough mixture, since more glutenins link together and form a heterogeneous mixture of polymers through disulfide bonded linkages of polypeptides [22]. For this reason, the presence of wheat flour in an enriched bread (with pumpkin seed, but also soya bean flour or others; [23,24]) is fundamental for the rheological characteristics of dough and, subsequently, of bread.

The highest water-soluble and salt-soluble protein fractions found in the pumpkin seed flour can affect chemical processes in the dough (proteolysis of proteins, changes in the redox potential in the dough, changes in the tertiary and quaternary structure of proteins), and its structural and mechanical characteristics [24].

The high biological value of pumpkin seed flour is due to a higher presence of EAA than that found in the wheat flour (Table 2).

The analysis of EAA showed a significantly higher EAA concentration in pumpkin seed flour when compared with the wheat flour. The predominant essential amino acid of pumpkin seed flour was leucine, whereas tryptophan was the lowest. This composition was confirmed by other authors [25], even though they found a higher concentration of all EAA when compared with the results of the present work.

The functional and technological properties of flours determine their behavior during processing, and characterize the ability to bind and retain moisture [26]. Indeed, these indicators influence the ability to form a viscoelastic dough, which is important in obtaining high quality bread, and provides its necessary structure and technological properties. To develop the required characteristics of the final product, the functional and technological properties of pumpkin seed flour were analyzed (Table 3).

Both moisture binding capacity and moisture retaining capacity were significantly higher in pumpkin seed flour than in wheat flour due to the higher content of fiber generally present in pumpkin seed [7]. This aspect results as positive in the dough and in the bread, since fiber and high moisture can positively influence the softness of grain-based products.

### 2.2. Near-Infrared Reflection Spectra of Flours and Doughs

**Figure 2:**
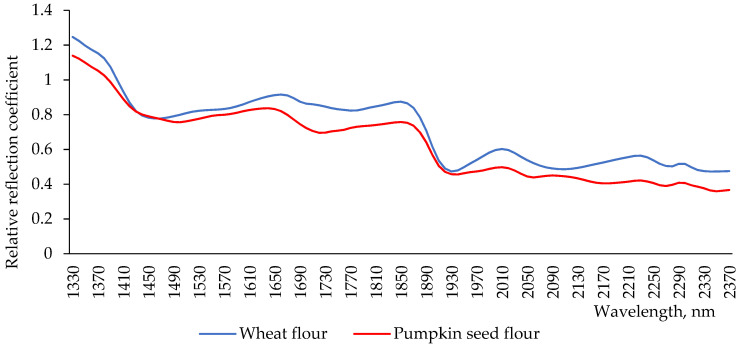
Spectra of reflection of wheat flour and pumpkin seed flour.

Flour is a material which consists of a set of particles. Their size exceeds the wavelength of light. Therefore, there is a propagation of optical radiation in all directions (Tyndall effect), which forms a white reflection. To identify and analyze the components of such materials, it is advisable to use the reflection spectrum in the near infrared area [27]. The reflection spectra of wheat flour and pumpkin seed flour showed little differences (Figure 2). In the first part of spectra, the minimum reflection for the wheat flour sample was observed at a wavelength of 1460 nm, and for pumpkin seed flour, it was shifted to 1500 nm. In the wavelength range 1440–1480 nm, the spectra of both flours coincided. This aspect indicates approximately the same water content in their chemical composition [28]. At the wavelength of 2100 nm, which characterizes the content and behavior of starch [29], there was an interesting feature: both spectra had extremes, but for pumpkin seed flour, this was the maximum extreme, and, for wheat flour, this was the minimum extreme of the intensity of the reflection. Indicators at this wavelength showed the presence of starch [29]. This difference in extremes indicates that wheat flour has higher starch content than pumpkin seed flour. Near this region, in the spectrum of pumpkin seed flour, two other additional minima of intensity were observed at 2260 and 2180 nm. The intensity of reflection at 2180 nm indicates the presence of total proteins [30]. Their content in pumpkin seed flour was higher than in wheat flour, correlating with values of the total protein content in the investigated types of flour (Figure 1). The different intensity of reflection at this wavelength can be explained by the difference in the fractional composition of proteins of the studied types of flour, and by the different content between soluble and insoluble proteins in the flours under investigation (Table 1). The last extreme in the spectrum of pumpkin seed flour was observed at the wavelength of 2345 nm, which indicates a high content of dietary fiber in it [31].

**Figure 3:**
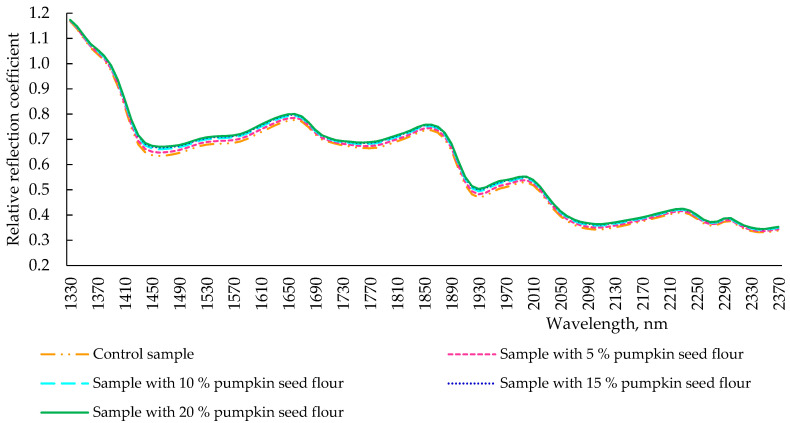
Spectra of reflection of dough samples (after kneading) with pumpkin seed flour at different concentrations (0, 5, 10, 15, and 20 %).

After kneading the dough, the spectra of the obtained samples with different concentrations of pumpkin seed flour to replace part of the wheat flour differed slightly, even though all the extremes were identical (Figure 3). However, the spectrum of the control sample had a lower intensity of reflection than pumpkin seed flour-addicted samples, and an evident pumpkin seed flour-dependent intensity of reflection was observed in the other samples. At 1930 nm, the wavelength responsible for the moisture content in the product, the reflection intensity of all samples coincided. This could be due to the fact that the samples were dried. The similar nature of the obtained spectra indicated that the determining factor influencing the formation of the properties of the dough was wheat flour, since it was the mass fraction which remains the largest in the dough. However, during the formation of the dough, there were changes in the structural elements (in particular, the absorption of water by fiber contained in pumpkin seed flour) in dough with pumpkin seed flour when compared with control sample. For this reason, the intensity of the reflection of pumpkin seed flour, independently of the pumpkin seed flour percentage, tended to the spectrum of control dough, acquiring springy-elastic and viscous-plastic properties that are conferred in the control sample by the content of glutenins and gliadins of wheat flour.

**Figure 4:**
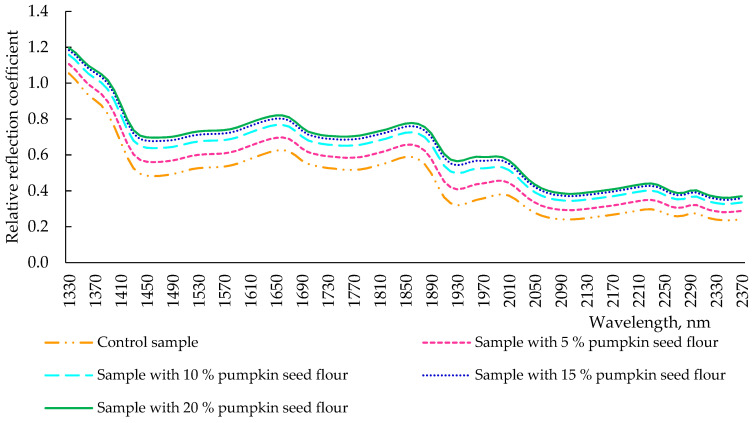
Spectra of reflection of dough samples (after 3.5 h of fermentation) with pumpkin seed flour at different concentrations (0, 5, 10, 15, and 20%).

The total time of fermentation and keeping of the dough is 3.5 h. The conformational changes in the structural substances of the dough were monitored after the completion of the fermentation (Figure 4). After kneading (3.5 h), all spectra of dough samples with different dosages of pumpkin seed flour changed their reflection intensity, but all extremes remained at the wavelength values observed in Figure 3. There was a clear pattern: the intensity of the reflection spectrum increased in a pumpkin seed flour-dependent way, and the control sample had the lowest location of its spectrum. The nature of the spectra of the dough of all samples after fermentation repeated the spectrum of wheat flour (Figure 2), but the intensity of the reflection of the samples tended to pumpkin seed flour. This was noticeable, especially at the maximum dosage of pumpkin seed flour (20%). This was due to the fact that the additive pumpkin seed flour contained much more dietary fiber in its composition, as reported for pumpkin seed flour-fortified muffins in the experiment conducted by Białek et al. [9], which observed an increasing fiber content dependent on the pumpkin seed flour concentration. Moreover, these changes in the reflection intensity can also be due to the redistribution of water in pumpkin seed flour dough samples, which led to partial dehydration of the gluten network, already reduced by the lower glutenin content in pumpkin seed flour when compared with wheat flour, and, thus, to the modifications of protein secondary structures [32]. Indeed, a loss of elastic α-helix structures was carried out, reducing the viscosity and the elasticity of the dough samples [32].

Comparing the control dough sample analyzed immediately after kneading (Figure 3) and after 3.5 h of fermentation (Figure 4), these samples had a similar nature, but a different intensity of reflection. Indeed, after the fermentation process, the reflection intensity of the dough decreased (Figure 3 and Figure 4). This was because, during fermentation, microbiological and biochemical processes occurred in the dough, and the hydration of gluten took place [33]. The wheat flour dough generally reveals a protein secondary structure characterized by α-helix conformations, which, during the fermentation process, helps to capture carbon dioxide bubbles, intensifying the gas formation and gas retention [14,34]. As already specified, adding pumpkin seed flour led to a gluten reduction in the dough and, thus, to lower hydration of the gluten network when compared with the control dough and, consequently, to a loss of the α-helices in the protein structure, reducing its viscosity and elasticity.

**Figure 5:**
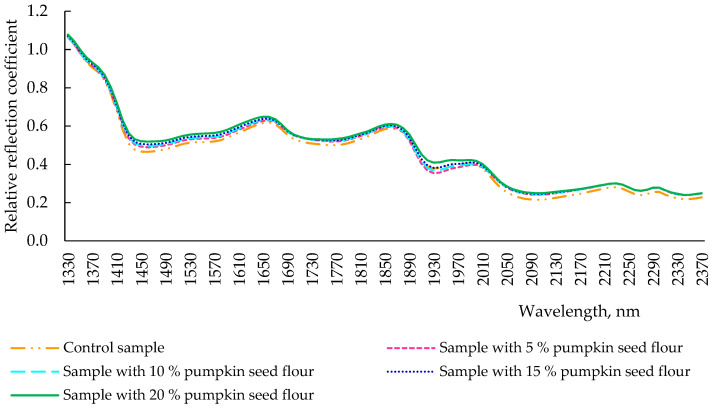
Spectra of reflection of bread with pumpkin seed flour at different concentrations (0, 5, 10, 15, and 20%).

During the process of baking, under the influence of high temperatures (220 °C), changes in protein and other substances of the product occurred, uniforming the reflection intensity of the final products (Figure 5). Indeed, throughout the analyzed wavelength range (1330–2370 nm), the reflection spectra of the control sample of the bread and samples with different dosages of pumpkin seed flour resulted very similarly. However, the control sample had a slightly lower reflection intensity throughout the spectrum when compared with the additive bread samples. This might be due to the lower presence of α-helical structures, which are one of the structural elements responsible for the viscosity of the dough, in the control sample and in the samples with pumpkin seed flour addition.

A variety of functional groups (carbonyl, hydroxyl, carboxyl, amide, amino, and cyano) can be quickly and reliably identified using infrared spectroscopy. In particular, most food products contain OH, CH, and NH functional groups, which are determined by the formed spectra (by analyzing the frequency and amplitude). Spectra arising from the oscillations of various groups in the near-infrared region have characteristic bands and characteristic frequencies [35]. In the near-infrared region, overtones of normal oscillations are used to analyze food products and technological mixtures [35]. The spectral index lg (1/R) was used to assess food quality parameters.

**Figure 6:**
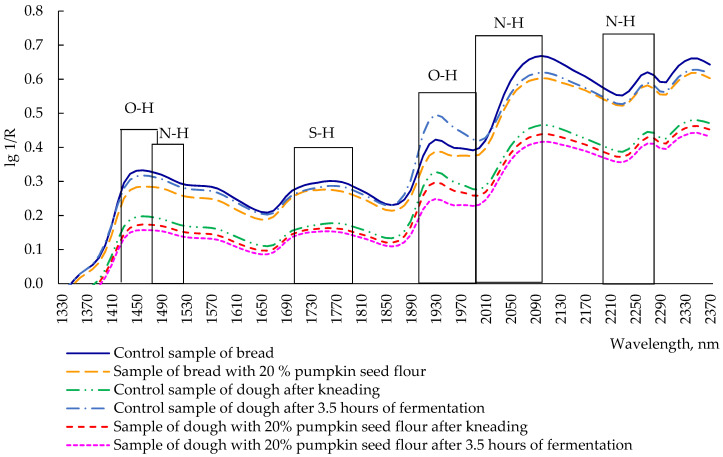
Changes and redistribution of structural groups in bread and dough after kneading and after 3.5 h of fermentation.

Analyzing the reflection spectra of the dough after kneading, after 3.5 h of fermentation, and of bread (both control sample and sample with 20% pumpkin seed flour), the highest reflection was noted at wavelengths of 1460 nm, 1760 nm, 1930 nm, 2100 nm, 2270 nm, and 2350 nm (Figure 6), with the extremum for all analyzed samples at 1460 nm, corresponding to the first overtone of valence vibrations of the OH group [36]. The functional groups of the first overtones, C-H and S-H, appear at 1760 nm [36]. Water is characterized by reflection at 1930 nm (OH deformation oscillations, the second overtone) [36]. To determine the content of gluten, the wavelength of 2100 nm was used, which corresponds to the second overtone of N-H deformation vibrations [36]. The wavelength of 2270 nm (O-H valence vibrations/C-O valence combination) is characteristic of lignin [36]. The maximum reflection at 2350 nm (the second overtone of C-H deformation vibrations) is responsible for the presence of lipids, which give products an oily taste [36].

The evaluation of the protein content in food products is usually carried out at 1510 nm (N-H valence vibrations, first overtone), 2060 nm (N-H deformation vibrations, second overtone or N-H deformation/N-H valence combination), and 2180 nm (N-H deformation oscillations, second overtone); however, these spectra did not show any extreme reflection.

The spectrum of the control sample of bread was the highest among all samples, except at 1930 nm, where the control sample of dough after 3.5 h of fermentation reported a reflection intensity of 0.5 lg 1/R. Bread with 20% pumpkin flour had a slightly lower intensity than the control bread, but, in some areas, both the spectra were overlapping, in particular at 1730, 2050, and 2250 nm. The other three spectra (the control sample of the dough after kneading, and the dough with 20% pumpkin seed flour after kneading and after 3.5 h of fermentation) were located in a separate group with lower reflection intensity when compared with bread samples and control dough after fermentation. The spectra of the dough with 20% pumpkin seed flour had the lowest reflection intensity among all samples, but the sample after kneading was located above the fermented sample. Regardless of the type of used flour, the samples of bread had a higher intensity of reflection than dough. This can be explained by the effect of high temperature in the baking process, which causes significant changes in protein and starch, fat, and other substances [37]. Indeed, the baking process can lead to damaged starch, through the formation of swollen granules or amylase leaching, affecting the rheological properties of dough and, consequently, the interaction with other chemical compounds [38]. Regarding the protein content, high baking temperatures and the low moisture percentage can lead to the aggregation of gluten polymers (in the case of wheat bread), or to an influence of sugars of the little amount of gluten present in pumpkin seed bread [39].

### 2.3. EAA Composition and Biological Value of Bread

The EAA profile of bread with different percentages of pumpkin seed flour is reported in Table 4. The results showed an increase of EAA dependent to the increase of pumpkin seed flour added to the wheat flour in the bread preparation. These results agree with the findings of other authors that tried the enrichment with pumpkin seed flour and other vegetable flours [40,41]. For example, Carlson et al. [40] reported that supplementation of the wheat flour with 10–20% tomato seed flour increased lysine content by 40–69% in breads, and El-Souccary [41] observed an increase of sulfur amino acid in enriched bread with pumpkin seed flour, especially after the roasting of bread.

Finally, the utilitarian coefficient of the EAA composition, characterizing the balance of EAA in relation to the physiologically necessary norm, was calculated (Table 5). To characterize the total mass of EAA for anabolic needs in human beings, the indicator of comparative excess of EAA (redundancy coefficient) was calculated, and the average value of excess EAA composition (an indicator of the coefficient of difference of amino acid score—DCAS indicator) and biological value were also determined (Table 5).

Analysis of the utilitarian coefficient, redundancy coefficient, and biological value of bread enriched with different percentages of pumpkin seed flour indicates a similar balance of EAA in proteic structures in all samples. However, as the percentage of pumpkin seed flour enrichment increased, the DCAS decreased, subsequently increasing the use of EAA by the human body, which was dependent on the pumpkin seed flour percentage.

## 3. Materials and Methods

### 3.1. Preparation of Flour, Dough, and Bread Samples

Protein composition and functional and technological properties (total protein content, protein fractional composition, essential amino acid composition, moisture binding capacity, and moisture retaining capacity) of premium wheat flour and pumpkin seed flour were analyzed following the methods described in paragraphs 2.2–2.5 and compared. During the flour preparation, 500 g seeds of wheat and of pumpkin bought in a local market were milled in a heavy grinder, sifted through a mesh sieve (200 μm aperture size) to obtain a fine powder, and kept at 4 °C until further use. Then, dough samples were prepared, each with 200 g premium wheat flour, 5 g salt, 7 g pressed baker’s yeast, 300 g water, and pumpkin seed flour in the amount of 5%, 10%, 15%, and 20% to replace wheat flour, and the prepared dough samples were fermented for 3.5 h at room temperature (25 °C). A sample without pumpkin seed flour was used as a control sample. After the investigation of semi-finished products, bread from doughs prepared with different concentrations of pumpkin seed flour and wheat flour for the control sample was prepared, cooking at 220 °C for 25 min in a ventilated oven (Sveba-Dahlen AB, Fristad, Sweden). Three breads for each treatment (each from 500 g of dough) were obtained and stored at room temperature (25 °C) for one week, until the analysis. Flours were analyzed for total protein content, mass functional composition of protein, essential amino acid profile, and their biological and technological value. The reflection spectroscopic profile of doughs was analyzed immediately after kneading and after 3.5 h of fermentation. Finally, the bread was analyzed spectroscopically for its EAA composition and biological value.

### 3.2. Total Protein Content

Flour mineralization with a strong acid releasing nitrogen can determine N content by the titration technique, following the Kjeldahl method. An amount of 1 g of raw material was hydrolyzed with 15 mL concentrated sulfuric acid containing two copper catalyst tablets for 2 h in a heat block at 420 °C. After cooling, distilled H_2_O was added to the hydrolysates before neutralization and titration [42]. The amount of present protein was calculated from the nitrogen concentration in the product. Total N was expressed as g proteins per 100 g of flour.

### 3.3. Protein Fractional Composition

The seed flour was defatted with pentane. The flour/solvent slurry was stirred at a 1:10 *w*/*v* ratio for 24 h, and the solvent was then removed by centrifugation. The mixture was dried at room temperature and stored in airtight sample bottles at 4 °C until subsequent use. Then, pumpkin proteins were fractionated from pentane defatted meal according to the Osborne differential extraction procedure described by Horax et al. [43]. The meal/water suspensions (20 g of meal into 100 mL of deionized water) were stirred for 2 h at room temperature and centrifuged for 30 min at 20,000× *g* to separate the supernatant from the pellet, thus obtaining the deionized water extract (DWE). The same extraction/separation conditions were kept for the next successive protein extraction steps. The water extract pellet was resuspended into 100 mL of 1 M NaCl solution and stirred as mentioned above. The supernatant obtained after centrifugation, designated as salt extract (SE), was then extracted in 100 mL of deionized water adjusted at pH 11 with 0.5 M NaOH solution, leading to the alkaline extract (AE). Each extraction was repeated twice. After each extraction, the pellets were washed twice using 20 mL of solvent to collect the residual protein entrapped in the insoluble residues. The DWE, SE, and AE were precipitated for isolation by adjusting the pH of the obtained supernatant to the pH corresponding to the minimum of solubility (pHms) determined from the turbidity experiment [44]. The pH was adjusted by 1 M HCl or 1 M NaOH solutions in acidic or alkaline pH. After centrifugation at 15,000× *g* for 15 min, the isolated protein precipitates were washed twice using deionized water at their respective pHms and recentrifuged. Finally, the resulting protein fractions were resolubilized by adjusting the pH to 7.0, freeze-dried, and stored at 4 °C until further analysis [45].

### 3.4. Essential Amino Acid (EAA) Composition

The determination of amino acid composition was conducted in accordance with the method of ion exchange chromatography [22]. The quality and quantitative determination of amino acids consisted of the hydrolysis of proteins and the determination of their quantitative estimation with the help of an automatic analyzer of amino acids, T-339 (Mikrotechna Praha a.s., Praha, Czech Republic), using polystyrene sulfonate ion exchange resins of “Ostion LJ ANB” in Li-citrate buffer one-column mode. The elution of amino acids from the column was conducted, in turn, by Li-citrate buffers from pH 2.75 ± 0.01; pH 2.95 ± 0.01; pH 3.2 ± 0.02; pH 3.8 ± 0.02; pH 5.0 ± 0.2. Amino acids were detected at a wavelength of 560 nm by rectification with a ninhydrin solution on a photometer (Unicam SP 800, Unicam Instruments, Cambridge, Britain). The results of detection were registered by a variplotter in the form of the peaks of absorption of light of ninhydrin-positive substances in an eluent that number the direct ratio concentrations of this substance in solution. The correlation of the solution of ninhydrin reagent and eluents was 1 to 2; the temperature of thermostatic T1 = 38.5 °C; T2 = 65 °C. The prototype was diluted in Li-citrate buffer by pH 2.2 ± 0.02 and inflicted on an ion exchange column. The quantitative estimation of chromatograms of the pre-production model settled in relation to the Bio-Rad standard mixture of amino acids. The mass of every amino acid, expressed as g per 100 g protein (Ai), in the investigated solution was calculated by the following formula:

$$\mathrm{Ai}=\frac{M_{i}\cdot S_{i}}{S_{e}^{3}}$$

where *M_i_* is molecular mass of each amino acid; *S_i_* is an area of peak of each amino acid on an aminogram from the investigated solution; *S_e_* is an area of peak of each amino acid on an aminogram from the solution of the standard mixture of amino acids which accords to one micromole.

Amino acid SCORE is expected according to the certificate scale of THEO/WHO [46].

### 3.5. Moisture Binding and Retaining Capacity

Moisture binding capacity was determined by the centrifugation method [47], and was calculated as follows:

Moisture binding capacity (%) = (weight of precipitate/weight of original flour) × 100

The determination of the moisture retaining capacity was carried out by mixing the sample with water and determining the amount of separated liquid after a centrifugation [48], and was calculated as follows:

Moisture retaining capacity (%) = [(weight of tube with flour and water retained − weight of tube with flour)/(weight of flour)] × 100

### 3.6. Near-Infrared Reflection Spectroscopy

The reflection spectra from a smooth surface and shredded samples were researched by using an Infrapid spectrometer (Labor-Mim, Hungary) in the near-infrared range from 1330 to 2370 nm. The spectrometer initially recorded the reflectance spectrum from reference I0 (component part of the instrument); then, a reflection spectrum from the researched sample I was obtained. The spectra are represented as the reflectivity of R in relative units (the ratio of the intensities I/I0 = R), depending on the wavelength in nm [49,50,51]. The intensity of reflection was measured in wheat and pumpkin flours, in wheat dough immediately after kneading and after 3.5 h of fermentation, and in bread. The reflection intensity was expressed through the relative reflection coefficient.

### 3.7. Biological Value of Bread

The utilitarian coefficient (U) characterizes the balance of all EAA proteins with respect to the physiological norm. It is used to compare the protein composition of various food products based on their amino acid composition and the inadequacy of their use in the body. It was calculated by the formula:

$$U=\frac{C_{min}\cdot \sum_{j=1}^{8}A_{ej}}{\sum_{j=1}^{8}A_{j}}$$

where *C_min_* is *C_min_*—score of the first limited acid EAA units; *A_j_* is the mass fraction of the *j*-th of EAA in the product, mg/g protein; *A_ej_* is the mass fraction of the *j*-th of EAA in the protein model, mg/g protein, according to FAO/WHO scale.

The redundancy coefficient (σ_red_) shows the mass fraction of EAA in 100 g of the product, which is not fully used by the body. It was calculated by the formula:

$$\sigma_{\mathrm{red}}=\frac{\sum_{j=1}^{k}(A_{j}-C_{min}A_{ej})}{C_{min}}$$

DCAS is the average excess of EAA in comparison with the smallest amino acid score of the limiting amino acid. It was calculated by the formula:

$$\mathrm{DCAS}=\frac{\sum \Delta DAS}{n}$$

where Δ*DAS*—the difference in amino acid score for each EAA compared to the AS of a limiting amino acid expressed as %; *n*—the number of amino acids.

The biological value of protein (BV), expressed as % [52], was calculated by the formula:

BV=100−DCAS

### 3.8. Statistical Analysis

The results of the analyses of different flours (from wheat and from pumpkin seed) were compared with a two-tailed Student’s *t*-test using a significance level of 0.05. The results of the analyses of different breads were subjected to a one-way analysis of variance (ANOVA), using the different concentrations of pumpkin seed flour (0, 5, 10, 15, 20 %) as the factor of variation. All the means were separated by an LSD (Least Significant Difference) post-hoc test (*p* < 0.05). The normality of data was tested using the Shapiro–Wilk test, whereas the homoscedasticity was tested using Bartlett’s test. Data were expressed as mean ± standard deviation (SD). Statistical analysis was performed using GraphPad (GraphPad, La Jolla, CA, USA).

## 4. Conclusions

Conformational changes in the structure of protein substances of dough and bread from wheat flour occurred when adding pumpkin seed flour, due to their different protein compositions. The total content of proteins found in pumpkin seed flour was 40% lower than that of wheat flour, with a higher presence of EAA.

Near-infrared reflection spectroscopy confirmed the difference in the fractional composition of proteins of the studied flours, and the different content between soluble and insoluble proteins, as well as the presence of OH, CH, SH, and NH groups.

The protein profile was complete after the replacement of 10% or more of wheat flour with pumpkin seed flour; this could be because the score for all amino acids was higher than 100%. As the percentage of the substitution of wheat flour with pumpkin seed flour increases, the number of amino acids used for anabolic purposes decreases, and they are fully utilized by the human body. Further research must be conducted in order to determine the best content of pumpkin seed flour to introduce in wheat bread. Concerning further research to be carried out in light of the results of this research, taking into consideration the variations that different cultivars and phenotypes of pumpkin may have in terms of production and nutritional characteristics [53], from a food technology and marketing perspective, it would be interesting to investigate which specific types of pumpkins are more suitable for processing seeds into flour for baked products.

Furthermore, sensory acceptance analysis might explore the level of preference and the purchase intention of consumers in order to position pumpkin seed flour products in the right target market.

## Figures and Tables

**Table 1:** Fractional composition of proteins of wheat flour and pumpkin seed flour. Data were compared with Student’s *t*-test (*p* ≤ 0.05). Significance ***: *p* ≤ 0.001.

Mass Fraction of Proteins (g/100 g)	Wheat Flour	Pumpkin Seed Flour
Albumin	5.4 ± 0.21 ***	3.2 ± 0.11
Globulin	9.9 ± 0.34	68.3 ± 1.34 ***
Glutenin	27.1 ± 0.78 ***	19.9 ± 0.67
Prolamin	43.1 ± 1.12 ***	4.0 ± 0.16
Insoluble proteins	14.5 ± 0.46 ***	4.6 ± 0.19

**Table 2:** The content of essential amino acids (EAA) in wheat flour and pumpkin seed flour. Data were compared with Student’s *t*-test (*p* ≤ 0.05). Significance ***: *p* ≤ 0.001.

EAA (g/100 g of Flour)	Wheat Flour	Pumpkin Seed Flour
Valine	0.42 ± 0.01	1.58 ± 0.1 ***
Isoleucine	0.36 ± 0.01	1.28 ± 0.1 ***
Leucine	0.71 ± 0.02	2.42 ± 0.1 ***
Lysine	0.23 ± 0.01	1.24 ± 0.1 ***
Methionine	0.40 ± 0.01	0.60 ± 0.02 ***
Threonine	0.28 ± 0.01	0.99 ± 0.1 ***
Tryptophan	0.13 ± 0.01	0.57 ± 0.01 ***
Phenylalanine	0.52 ± 0.01	1.73 ± 0.1 ***

**Table 3:** Functional and technological properties of wheat flour and pumpkin seed flour. Data were compared with Student’s *t*-test (*p* ≤ 0.05). Significance ***: *p* ≤ 0.001.

Property	Wheat Flour	Pumpkin Seed Flour
moisture binding capacity (%)	90.7 ± 2.13	211.2 ± 3.21 ***
moisture retaining capacity (%)	148.0 ± 2.89	224.7 ± 3.75 ***

**Table 4:** Essential amino acid profile (EAA) of bread with the replacement of part of wheat flour with pumpkin seed flour at different percentages (0, 5, 10, 15, and 20%).

EAA (g/100 g of Bread)	Pumpkin Seed Flour (%)				
	0	5	10	15	20
valine	0.42 ± 0.01 e	0.58 ± 0.01 d	0.74 ± 0.02 c	0.89 ± 0.02 b	1.05 ± 0.05 a
isoleucine	0.70 ± 0.02 e	1.09 ± 0.1 d	1.48 ± 0.1 c	1.87 ± 0.1 b	2.25 ± 0.1 a
leucine	0.38 ± 0.01 e	0.52 ± 0.01 d	0.66 ± 0.01 c	0.79 ± 0.02 b	0.93 ± 0.03 a
lysine	0.30 ± 0.01 e	0.48 ± 0.01 d	0.66 ± 0.01 c	0.84 ± 0.02 b	1.02 ± 0.06 a
methionine	0.23 ± 0.01 e	0.37 ± 0.01 d	0.51 ± 0.01 c	0.64 ± 0.01 b	0.78 ± 0.02 a
threonine	0.66 ± 0.01 e	1.11 ± 0.1 d	1.56 ± 0.1 c	2.02 ± 0.1 b	2.46 ± 0.1 a
tryptophan	0.09 ± <0.01 e	0.18 ± <0.01 d	0.26 ± 0.01 c	0.35 ± 0.01 b	0.44 ± 0.01 a
phenylalanine	0.28 ± 0.01 e	0.64 ± 0.01 d	1.01 ± 0.05 c	1.37 ± 0.1 b	1.73 ± 0.1 a

Means (±SD; *n* = 3) indicated by different letters differ significantly (*p* < 0.05) following the one-way ANOVA with the different percentages of pumpkin seed flour as a variability factor.

**Table 5:** Utilitarian coefficient, redundancy coefficient, coefficient of difference of amino acid score (DCAS), and biological value of bread with the replacement of part of wheat flour with pumpkin seed flour at different percentages (0, 5, 10, 15, and 20%).

Indicator	Pumpkin Seed Flour to Replace Wheat Flour (%)					Significance
	0	5	10	15	20	
Utilitarian coefficient	0.49 ± 0.01	0.49 ± 0.01	0.48 ± 0.01	0.48 ± 0.01	0.48 ± 0.01	ns
Redundancy coefficient	36.90 ± 1.11	38.21 ± 1.17	38.83 ± 1.19	39.18 ± 1.19	39.41 ± 1.21	ns
DCAS	0.46 ± 0.01	0.80 ± 0.01	1.16 ± 0.01	1.53 ± 0.01	1.89 ± 0.01	ns
Biological value (%)	99.53 ± 2.49	99.19 ± 2.46	98.83 ± 2.43	98.46 ± 2.41	98.10 ± 2.36	ns

Means (±SD; *n* = 3) indicated by ns significance do not differ significantly (*p* > 0.05) following the one-way ANOVA with the different percentage of pumpkin seed flour as variability factor.

## Data Availability

Not applicable.
